# Changes in the TMS-evoked potential N100 in the dorsolateral prefrontal cortex as a function of depression severity in adolescents

**DOI:** 10.1007/s00702-022-02539-9

**Published:** 2022-08-27

**Authors:** Lea Biermann, Heidrun Lioba Wunram, Lena Pokorny, Eva Breitinger, Nicola Großheinrich, Tomasz Antoni Jarczok, Stephan Bender

**Affiliations:** 1 Department of Child and Adolescent Psychiatry, Psychosomatics, and Psychotherapy, University of Cologne, Faculty of Medicine and University Hospital Cologne, Cologne, Germany; 2 Catholic University of Applied Sciences North-Rhine-Westphalia/Cologne, Cologne, Germany; 3 Department of Child and Adolescent Psychiatry, Psychosomatics and Psychotherapy, University Hospital Frankfurt, Frankfurt, Germany; 4 Department of Child and Adolescent Psychiatry and Psychotherapy, KJF Klinik Josefinum, Augsburg, Germany

**Keywords:** TMS-EEG, N100, Depression, Adolescents, TEPs

## Abstract

Studies using transcranial magnetic stimulation with simultaneous electroencephalography (TMS-EEG) revealed an imbalance between cortical excitation and inhibition (E/I) in the dorsolateral prefrontal cortex (DLPFC) in depression. As adolescence is a developmental period with an increase in depression prevalence and profound neural changes, it is crucial to study the relationship between depression and cortical excitability in adolescence. We aimed to investigate the cortical excitability of the DLPFC in adolescents with depression and a dependency of the TMS-evoked potential N100 on the depression severity. 36 clinical patients (12–18 years of age; 21 females) with a major depressive episode were assessed twice in a longitudinal design: shortly after admission (T0) and after six weeks of intervention (T1). GABA-B-mediated cortical inhibition in the left and right DLPFC, as assessed by the N100, was recorded with EEG. Significantly higher depression scores were reported at T0 compared to T1 (*p* < 0.001). N100 amplitudes were significantly increased (i.e., more negative) at T0 compared to T1 (*p* = 0.03). No significant hemispheric difference was found in the N100 component. The correlation between the difference in depression severity and the difference in N100 amplitudes (T0–T1) obtained during stimulation of the left DLPFC did not remain significant after correction for testing in both hemispheres. Higher N100 amplitudes during a state of greater depression severity are suggestive of an E/I imbalance in the DLPFC in adolescents with an acute depressive episode. The N100 reduction potentially reflects a normalization of DLPFC over inhibition in association with decreased depressive symptomatology, indicating severity dependency.

## Trial registration

The main longitudinal controlled add-on sports intervention study was registered with the German Clinical Trials Register (DRKS ID: DRKS00011772).

## Introduction

Depression is the leading cause of disability worldwide. The associated risk of chronicity and suicidality emphasizes the need for effective treatment, in particular among youth (World Health Organization 2017). Identifying potential biomarkers of depression in adolescents is relevant in elucidating the pathophysiology of depression and enabling evaluations of treatment effects.

The dorsolateral prefrontal cortex (DLPFC) has been implicated in the neurobiology of depression, given its role in regulating negative emotions (i.e., reappraisal/suppression strategies) (Koenigs and Grafman 2009; Lévesque et al. 2003). Neuroimaging studies illustrated reduced DLPFC activity in patients with major depressive disorder (MDD) (Biver et al. 1994; Siegle et al. 2007), and the DLPFC is a prominent target for repetitive transcranial magnetic stimulation (rTMS) in the treatment of MDD (Daskalakis et al. 2008). Moreover, reduced levels of the excitatory neurotransmitter glutamate and the main inhibitory neurotransmitter gamma-aminobutyric acid (GABA) in MDD have been reported in the cortex in general and—more specifically—the prefrontal cortex (Hasler et al. 2007; Sanacora et al. 1999; Yüksel and Öngür, 2010). Particular significance in the pathogenesis of depression is attributed to the GABAergic system in the prefrontal cortex (Duman et al. 2019; Ghosal et al. 2017; Page and Coutellier 2019) and an imbalance between cortical excitation and inhibition (E/I) has been proposed as key mechanism (Krystal et al. 2002; Lener et al. 2017). Hence, investigating the E/I balance in the DLPFC, and more specifically the role of the GABAergic system, is important to further understand the pathophysiology of MDD.

While rTMS is used to induce changes in cortical excitability (Daskalakis et al. 2008; Grossheinrich et al. 2013), single-pulse TMS combined with electroencephalography (EEG) allows for the measurement of cortical excitability. Time-logged positive and negative EEG deflections following the TMS pulse are referred to as *TMS-evoked potentials* (TEPs) (Tremblay et al. 2019). A frequently studied TEP is the N100—a negative deflection approximately 100 ms after the TMS pulse (Bender et al. 2005; Komssi et al. 2004; Nikulin et al. 2003). The N100 reflects GABA-B mediated inhibitory processes (Premoli et al. 2014).

In cross-sectional studies of both adults and young adults, patients with MDD exhibited greater (i.e., more negative) N100 amplitudes in the DLPFC compared with healthy participants (Dhami et al. 2020; Voineskos et al. 2019), reflecting higher DLPFC inhibition. However, these between-group designs cannot elucidate whether N100 alterations in patients with MDD reflect a temporarily and situationally stable trait or a dependency on the severity of the depressive state. Severity-dependency of the N100 in adults with MDD has been indicated in a longitudinal study by reductions in N100 amplitudes consistent with a decrease in symptom severity after a six-week rTMS-EEG intervention (Voineskos et al. 2021). Correlations between symptom severity and N100 amplitudes in adults (Voineskos et al. 2019, 2021) suggests that the more severe the depressive symptoms, the higher the N100 amplitude (i.e., more negative), further emphasizing a dependency of the N100 on depressive symptoms severity. However, N100 amplitudes did not significantly change in young adults (16–24 years of age) with MDD, during a 2-weeks theta burst therapy (Dhami et al. 2021). The absence of an association between N100 amplitudes and clinical characteristics (Dhami et al. 2021, 2020) could instead indicate a N100 trait-dependency.

Neurobiological differences associated with developmental processes might explain contradictory N100 findings between adults and young adults with MDD (Zalsman et al. 2006). An imbalance in cortical maturation between earlier developing limbic systems (associated with emotional reactivity), and later developing prefrontal cortical control systems has been proposed (Casey 2015; Casey et al. 2008). Indeed, maturation of the DLPFC was observed as late as at the end of adolescence (Gogtay et al. 2004). Hence, age dependent refinements in organization and efficiency in the DLPFC define adolescence as a sensitive period (Casey 2015; Casey et al. 2001, 2008). An association between developmental DLPFC changes and the strong increases in the prevalence of depression onset from late childhood to young adulthood has been suggested (Davey et al. 2008). Additionally, the inhibitory GABAergic system undergoes extensive changes during adolescence (Caballero and Tseng 2016) and an association between GABA-B markers and age has been observed in children/adolescents with and without depression (Croarkin et al. 2014). Therefore, it is crucial to investigate the relationship between depression and the N100 in adolescence.

To our knowledge, this is the first study to examine the TMS-EEG evoked N100 in the DLPFC as a potential biomarker for MDD in adolescence in a pre-post-intervention design. We expected a significant reduction in depressive symptom severity during a six-week inpatient intervention and either no change in N100 amplitudes (trait marker) or a significant reduction in N100 amplitudes (marker for depression severity). We hypothesized a relationship between the change in depression severity and N100 amplitudes in case a reduction in N100 amplitudes was observed.

## Materials and methods

Data were collected as part of a longitudinal controlled add-on sports intervention study, in which participants received a 6-weeks sports therapy in addition to their treatment as usual. The main study was registered in the German Clinical Trials Register (DRKS- ID: DRKS00011772) and a study protocol has been published (Oberste et al. 2018). The experimental group conducted a whole-body vibrations training by performing static and dynamic exercises (e.g., squats, lunges) on the Galileo^®^ Whole Body Vibration Plate Med M (Novotec Medical GmbH, Pforzheim, Germany). The control group executed a myofascial training without muscular or cardiovascular strain, to control for psychosocial attention, by performing seven standardized exercises (e.g., self-massage of legs, arms and back) using a foam roll (Blackroll, Bottighofen, Switzerland). Please refer to the published study protocol for more information about all assessed parameters and details of the sport intervention (Oberste et al. 2018).

At the time of the interim analysis, subjects are still being recruited as part of the clinical trial. The therapeutic effects of the intervention will be analyzed when the clinical trial is completed (*N* = 82) and a sufficient number of participants per exercise group has been included (*n* = 41 per group) according to the a priori calculated power analysis (Oberste et al. 2018). However, in the interim analysis, we were not interested in examining the therapeutic effects of the sports intervention on the primary (depressive symptoms) or secondary (neurophysiological) endpoints. Instead, we focused on the fundamental relationship between depressive symptoms and the neurophysiological parameter N100 and changes in both parameters over time. Hence, data from both intervention arms (inpatient treatment as usual plus (a) add-on control-sports intervention or (b) add-on active-sports intervention) were collapsed for baseline (T0) and post-intervention (T1) measurements. Pooling the data from both exercise groups also results in a higher number of cases (*n* = 36) and thus a higher statistical power, which should be sufficient for the interim analysis after about half of the planned total sample size.

### Individuals and recruitment

Adolescent patients with MDD were recruited at the Clinic and Polyclinic for Child and Adolescent Psychiatry, Psychosomatics, and Psychotherapy, University Hospital Cologne by psychologists and psychiatrists. 38 participants completed T0 and T1 but two (~ 5%) TMS-EEG data sets contained non-removable confounding artifacts. Therefore, 36 participants entered the analysis (21 girls; mean age 15.5 ± 1.42 years).

Detailed inclusion and exclusion criteria are reported in the study protocol (Oberste et al. 2018). Inclusion criteria were diagnosis of a current depressive episode (see “Clinical assessment”), clinical treatment and age between 13 and 18 years. In accordance with the study protocol patients with acute substance abuse or addiction, a body mass index of < 16 kg/m^2^, acute suicidality, pervasive developmental disorder, IQ of < 70, schizophrenia, acute psychosis, or schizoaffective disorder were excluded. Conditions limiting sportive ability, Addison's disease, untreated hypothyroidism, and long-term medication with psychotropic drugs (e.g., neuroleptics) led to exclusion from study participation. Stable antidepressant medication for a period of at least six weeks before enrollment was accepted, provided that the medication continued unchanged during the study. Two (~ 5%) individuals had a stable antidepressive medication with Fluoxetine. Due to recruitment difficulties deviations from the study protocol were made. One participant was 12 years old at enrollment but turned 13 during inpatient treatment, and two participants were allowed to sleep at home and thus became day clinic patients who nonetheless received the same treatment as usual as inpatients. The duration of study enrollment was extended so that participants were enrolled within the first four weeks of clinical treatment instead of the first three weeks, provided they still met the inclusion criteria (see “Clinical assessment”). TMS safety guidelines were considered (Rossi et al. 2011).

### Clinical assessment

Individuals had a clinical diagnosis of MDD (F32.X and F33.X). ICD-10 criteria for a current depressive episode were confirmed via information assessed with the semi-structured clinical interviews *Children’s Depression Rating Scale Revised* (CDRS-R) (Keller et al. 2012) and the depression specific questionnaire *Depressionsinventar für Kinder und Jugendliche* (DIKJ) (Stiensmeier-Pelster et al. 2014) before enrollment. A raw score of ≥ 18 on the German DIKJ, which is based on the Children’s Depression Inventory (CDI) (Kovacs, 1992), further substantiated acute depressive symptoms (similar to the enrollment process in the conducted pilot study (Wunram et al. 2018)). The DIKJ corresponds to the MDD criteria of the DSM-5 (Stiensmeier-Pelster et al. 2014). Mean DIKJ score at T0 was 29.53 (SD = 7.13). Details of psychiatric assessments on axis-I comorbidity via the German version of the semi-structured clinical interview Kiddie Schedule for Affective Disorders and Schizophrenia—Present and lifetime version (K-SADS-PL) (Kaufman et al. 1997) are reported in the supplementary material (Online Resource Table A1).

Severity of depressive symptoms was assessed as primary outcome with the German version of the semi-structured clinical interview CDRS-R by blind raters. The CDRS-R follows the diagnostic criteria of the DSM-IV-TR (Keller et al. 2012) and is a common outcome measure in studies of MDD in adolescence (Plener et al. 2012; Richardson et al. 2014).

### Transcranial magnetic stimulation

Using a MagPro X100 with MagOption TMS stimulator (MagVenture, Farum, Denmark), biphasic single TMS pulses were applied to the left and right DLPFC by a figure-of-eight coil (MCF-B65, outer diameter 2 × 75 mm). The TMS clicking noise was not masked by earplugs/headphones, and no foam sheet was placed between the coil and the head. The coil was held by a trained investigator. The positions of EEG electrodes F5 and F6 were used as targets for stimulation of the left and right DLPFC, respectively. Studies have shown that this localization method is sufficiently accurate (Rusjan et al. 2010). Electromyogram data of the right first dorsal interosseous muscle (FDI) were recorded. The motor hot spot for the FDI was localized (Rossini et al. 2015) and the individual resting motor threshold (RMT) was determined by the maximum likelihood procedure (Awiszus and Borckardt 2006), using the Motor Threshold Assessment Tool (version 2.0: http://www.clinicalresearcher.org/software.htm).

### EEG recording

EEG signals were measured with a 64-channel BrainAmp system (BrainProducts, Munich, Germany) and recorded at a sampling rate of 5000 Hz in the Brain Vision Recorder software (BrainProducts). The TMS-compatible EEG cap (Easycap, Germany) has an equidistant montage corresponding to the extended 10–10 system and additional electrooculogram electrodes at the nasion and under both eyes. Cz was used as recording reference and impedances below 5 kΩ were ensured.

### Experimental protocol

The reported DLPFC stimulation at rest was part of a TMS experiment in which two other stimulation paradigms were performed. The other stimulation paradigms consisted of single-pulse stimulation of the right DLPFC during an emotional 1-back task and a paired-pulse protocol applied at M1. In this interim analysis, we focused on GABA-B effects in the DLPFC which is the primary outcome parameter for the TMS-EEG part of our study. In addition, the signal-to-noise ratio is lower in the task paradigm due to a lower number of TMS pulses per emotional condition in the N-back task (sadness, neutral, happiness). The N100 effects in the task condition (lower signal-to-noise ratio) and in M1 (paired-pulse secondary parameters) will be analyzed when the clinical trial has been completed, and the larger sample yields higher statistical power for these analyses. Analyses of these data have not yet been performed.

During the resting condition, participants visually fixated a cross on a screen. Presentation software 18.1. (NeuroBehavioral Systems, Berkley, USA) was used for a standardized application of TMS pulses. Forty-five TMS single pulses were applied in blocks to the left and right DLPFC in counterbalanced order with randomized stimulus intervals (5–8 s). Good signal-to-noise ratios (SNR) have been obtained for N100 peaks with ~ 45 and ~ 48 trials in previous TMS-EEG studies (Chung et al. 2017, 2018). In combination with a suprathreshold stimulation intensity of 120% RMT, a good SNR can be expected with 45 trials, especially in children and adolescents, as they show high N100 amplitudes (Bender et al. 2005).

### Data analysis

#### Signal pre-processing

BrainVision Analyzer (BrainProducts, Munich, Germany) was used to analyze EEG data similar to previously published procedures (Roos et al. 2021). The large file size was reduced by downsampling to 500 Hz. The downsampling process with the implemented anti-aliasing filter (low-pass-filter 225 Hz) may slightly distort the TMS pulse artifact (Rogasch et al. 2017), but we verified that the examined time window (80–140 ms) was not affected. EEG data were interpolated − 10 to 20 ms around the TMS pulse to remove the TMS pulse artifact. Data were referenced to an average reference and EEG data were segmented into epochs of − 500 to 500 ms around the TMS pulse. Severe noise (e.g., large muscular artifacts) was manually rejected in individual channels and data were baseline corrected (− 110 to − 10 ms). Blink and eye movement artifacts were removed by independent component analysis (Ilmoniemi et al. 2015). Linear DC detrending and a 50 Hz notch filter were applied. An inspection revealed that the DC detrend did not change grand averages systematically but reduced variance. Averages were calculated for left and right DLPFC stimulation condition for each measurement (T0/T1).

#### N100 analysis

The TMS-evoked N100 maximum has been described ipsilaterally over the stimulation site (Bonato et al. 2006; Jarczok et al. 2021). Clearly lateralized ipsilateral TEPs at the stimulation site likely reflect genuine cortical activity and not merely activity due to peripheral stimulation (Conde et al. 2019). Therefore, ipsilateral electrodes F5 and F6 were selected as electrodes of interest for the left and right DLPFC stimulation condition, respectively (Ilmoniemi and Kičić 2010). Similar to previous studies, the N100 was defined as the highest negative peak in the 80–140 ms time interval at the electrode of interest (Kerwin et al. 2018; Rogasch et al. 2014, 2015). Mean amplitudes ± 10 ms around the peak were exported.

To systematically examine lateralized activity, i.e., transcranially evoked cortical activity at the stimulation site that depends on the side of stimulation (left/right), a calculation analogous to the lateralized readiness potential (LRP) (Coles, 1989) was performed. This procedure integrates information from both ipsilateral and contralateral homologous electrodes for each stimulation condition, eliminating symmetric activity that is not specific to the stimulation condition. Similar to a previous study in our group (Jarczok et al. 2021), a single measure called LatTEP N100 was calculated based on the TEPs of the homologous electrodes. The calculation is performed according to the following example procedure (Coles, 1989): LatTEP N100 F5/F6 = [F5(TMS left) − F6(TMS left) + F6(TMS right) − F5(TMS right)]/2. This formula was applied to each homologous electrode pair, resulting in a topographic plot on one side for each time point, which contains information on the lateralized activity of both stimulation conditions (left/right). The peak for the LatTEP N100 component was placed in the 80–140 ms time window in the reference channel F5/F6, and mean amplitudes ± 10 ms around the peak were exported.

### Statistics

IBM SPSS Statistics 28 (IBM Corp., Armonk, NY, USA) was used for statistical analysis. Shapiro-Wilks tests revealed a normal distribution of CDRS-R values at both time points and a normal distribution of CDRS-R and N100 amplitude difference values (T0–T1). The normality assumption was neither met for all N100 amplitudes in each stimulation condition (time point × stimulation site), LatTEP N100 F5/F6 amplitudes at T0 and T1 nor for age. The N100 amplitudes of each stimulation condition were nonetheless entered into a two-way repeated-measures analysis of variance (rANOVA) because it is robust against violations of normality and, as a parametric method, has the advantage of higher statistical power compared with nonparametric methods (Blanca et al. 2017; Schmider et al. 2010). Nonparametric methods were used for comparisons between LatTEP N100 F5/F6 amplitudes at T0 and T1 as well as correlational analysis between N100 amplitudes of each stimulation condition and age and sex, due to the violation of normality.

Outlier analysis revealed two extreme outliers of more than ± 3 standard deviations (SD) from the mean (*M*) N100 amplitude during stimulation of the left DLPFC at T0. In this condition, 90% winsorization was performed by replacing the upper and lower 5 percent of the N100 amplitudes with the value of the 5th and 95th percentiles, respectively, to avoid distortions by outliers (Leys et al. 2019; Tukey and McLaughlin 1963).

Reductions in depressive symptom severity, as assessed by CDRS-R scores at T0 and T1, were examined by a dependent t-test. Two-way rANOVAs with TIME (T0/T1) and STIMULATION SIDE (left stimulation at electrode F5/right stimulation at electrode F6) as within-subject factors were used with the dependent variable N100 amplitudes to test our hypothesis on N100 amplitude alterations. A nonparametric Wilcoxon signed-rank test was applied to test the difference in LatTEP N100 amplitudes at F5/F6 between T0 and T1. To estimate the effect size, the *z*-score was normalized by sample size, i.e., *r* = *z*/$\sqrt{N}$ (Rosenthal 1991).

Two correlational analyses were performed: Spearman’s correlation coefficients were calculated between N100 amplitudes and age as well as sex, because the N100 amplitudes in each stimulation condition (time point × stimulation site) were not distributed normally. Since the N100 and CDRS-R difference values were distributed normally, parametric Pearson correlations between CDRS-R difference values and N100 amplitudes difference values (T0–T1) were evaluated by one-sided tests according to the hypothesis. For multiple comparisons, *p*-values were Bonferroni-Holm corrected and a statistical significance level of *p* < 0.05 was applied.

## Results

### Depressive symptoms

A dependent t-test demonstrated a signficiant decrease in depressive symptom severity by higher CDRS-R scores at T1 (*M* = 44.83, SD = 13.15) compared with T0 (*M* = 54.97, SD = 13.71) (difference: *M* = 10.14, *SD* = 11.36; *t*(35) = 5.35, *p* < 0.001, *d* = 0.89).

### N100 amplitudes and difference values

**Fig. 1:**
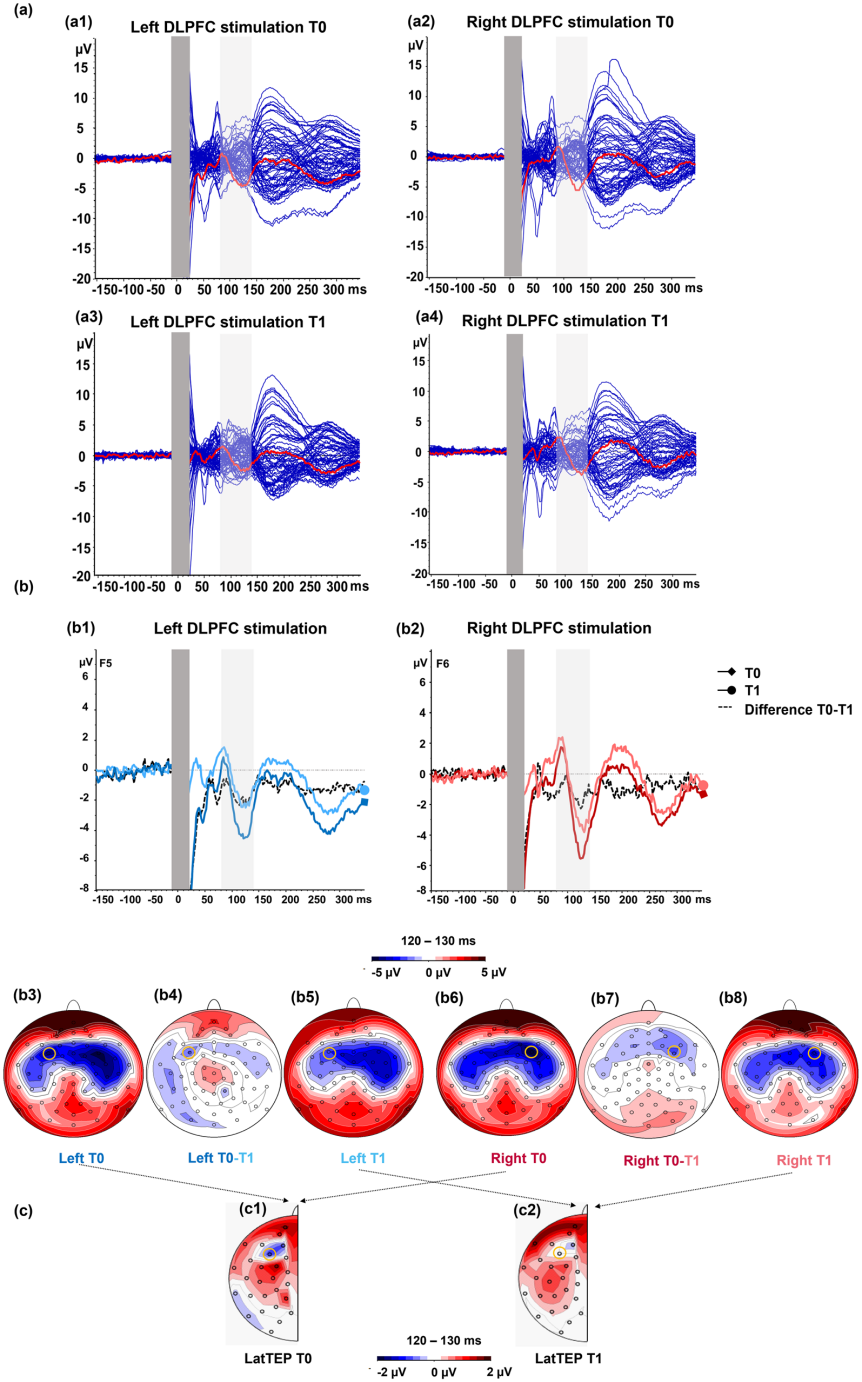
Group grand average TEP amplitudes and N100 topographies. Butterfly plots (a) are illustrated for each stimulation condition. Red lines indicate the TEP time course at electrode F5 during left DLPFC stimulation (a1 and a3) and at F6 during right DLPFC stimulation (a2 and a4). TEP time courses for each time point (T0, T1) as well as difference waves (T0–T1) are displayed for the left (b1) and right (b2) stimulation condition at the respective electrode of interest (F5 and F6). Dark blue and dark red lines (squared arrowheads) indicate amplitudes measured at baseline (T0). Lighter blue and lighter red lines (round arrowhead) indicate amplitudes measured post-intervention (T1). Dotted lines indicate the difference wave between T0 and T1. Light gray bars highlight the time window (80–140 ms) used for the N100 peak detection. The TMS artifact (dark gray bars) has been cut out. The topographical maps illustrate the activity during left (b3–b5) and right (b6–b8) stimulation at T0 and T1 as well as the difference between T0 and T1. To illustrate N100 topographies the time window 120–130 ms was chosen, based on peaks in the grand average. Variations in individual peak width explain earlier mean peak latencies based on single subject averages compared with peak latencies displayed in grand averages. Lateralized activity maps for T0 (c1) and T1 (c2) illustrate TMS‐evoked cortical activation, in which symmetrical activity between hemispheres is subtracted. As a result of the calculation between homologous electrodes, one channel remains (depicted arbitrarily on the left side of the head), which encompasses signals from both stimulation sites (left and right stimulation) and both hemispheres. Yellow circles mark the position of electrodes of interest (F5/F6) in the topographies. Please note that the original data maps are plotted on 5 μV scale while lateralized activity was plotted on 2 μV scale

**Fig. 2:**
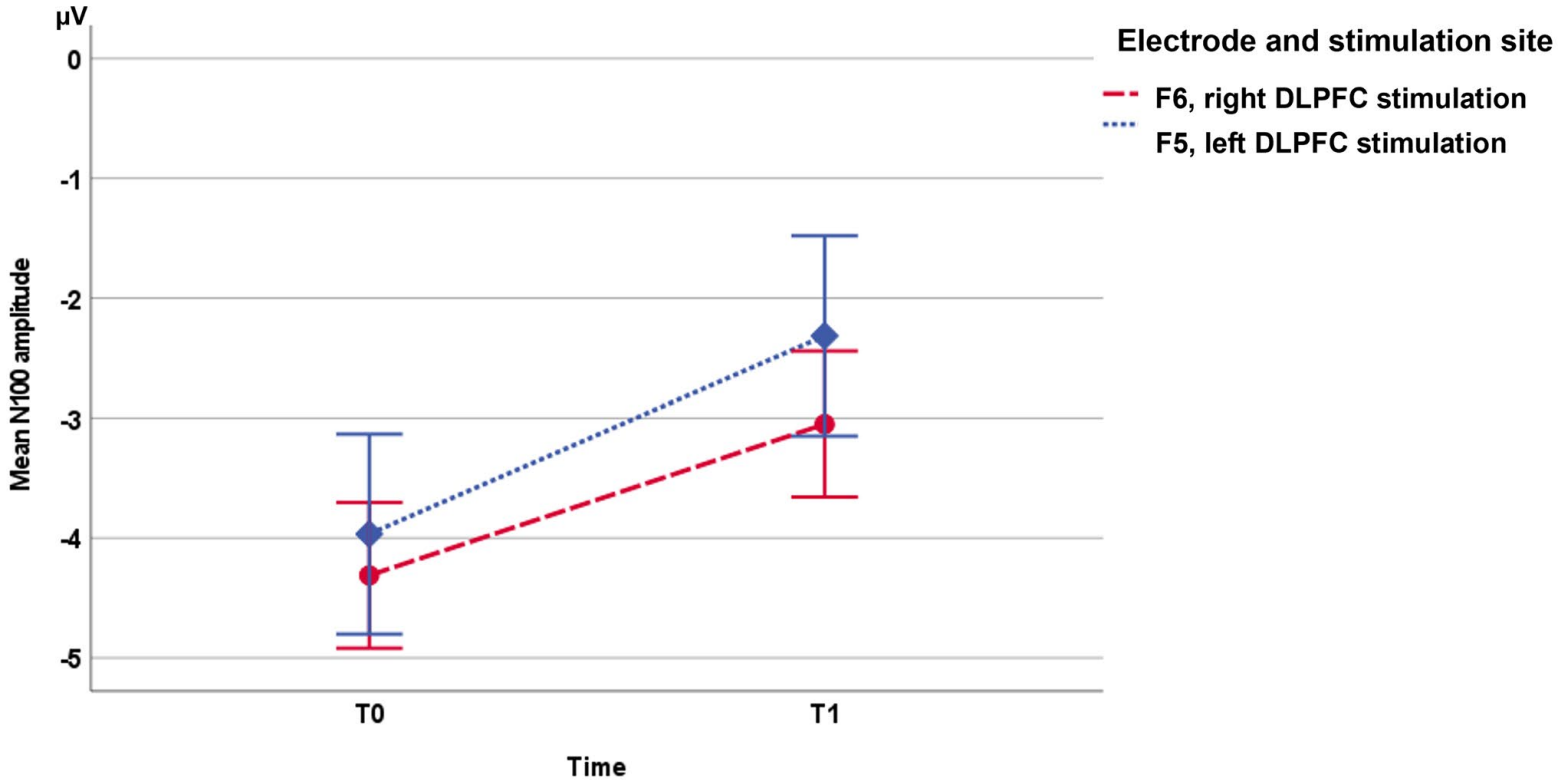
Error bar chart of the mean N100 amplitudes at the ipsilateral electrode of interest during left (F5) and right (F6) DLPFC stimulation at baseline (T0) and post-intervention measure (T1). Error bars reflect 95% confidence intervals adapted to repeated-measures designs (Cousineau 2005)

A rANOVA with the dependent variable N100 amplitude returned a significant main effect for the within-subject factor TIME (Table 1), with larger N100 amplitudes at T0 measurement (Figs. 1 and 2).

**Table 1:** RANOVA with dependent variable N100 amplitudes

Effects		N100 amplitudes [µV]			
		*F*	df	*p*	*η*^2^_p_
Main	Stimulation side	1.67	1, 35	0.21	0.05
	Time	5.15	1, 35	0.03*	0.13
Interaction	Stimulation side × time	0.31	1, 35	0.58	0.01

*F* test statistic, *df* degrees of freedom, *p* two-tailed significances, *η*^*2*^_*p*_ effect size partial eta squared

*p < 0.05

Descriptive statistics on N100 amplitudes and latencies are provided in Table 2. No significant correlations between N100 amplitudes (scores at T0, T1 and difference values) and age or sex were found (see Online Resource Table B1).

**Table 2:** N100 descriptive statistics

Condition	Amplitude [µV]				Latency [ms]			
	*M*	SD	Mdn	IQR	*M*	SD	Mdn	IQR
Electrode F6, right DLPFC stimulation at T0	− 4.31	4.68	− 3.29	− 6.47 to − 0.95	120.72	12.53	124.00	114.00–130.00
Electrode F6, right DLPFC stimulation at T1	− 3.05	3.55	− 2.21	− 6.34 to 0.03	122.00	12.62	126.00	116.00–130.00
Electrode F5, left DLPFC stimulation at T0	− 3.96	5.21	− 2.31	− 5.04 to − 0.60	118.83	13.01	121.00	114.00–128.00
Electrode F5, left DLPFC stimulation at T1	− 2.31	2.94	− 2.13	− 3.89 to − 0.24	114.50	14.99	118.00	101.50–127.50
LatTEP N100 F5/F6 T0	− 1.82	4.49	− 0.58	− 2.60 to 0.45	113.00	16.09	113.00	97.50–128.00
LatTEP N100 F5/F6 T1	− 0.53	2.78	− 0.38	− 1.77 to 1.34	110.44	16.75	112.00	94.50–126.00

*M* arithmetic mean, *SD* standard deviation, *Mdn* median, *IQR* interquartile range (IQR: 25–75%)

### LatTEP N100 F5/F6 amplitudes

The Wilcoxon signed-rank test revealed a trend toward significantly higher LatTEP N100 amplitudes at electrode F5/F6 during the T0 measurement compared with the T1 measurement (*Z* = − 1.82, *p* = 0.07, *r* = − 0.30). Descriptive statistics on LatTEP N100 F5/F6 amplitudes and latencies are provided in Table 2.

### Association between depressive symptoms and N100

Pearson correlations between CDRS-R difference values and N100 amplitude difference values (T0-T1) were significant for the left DLPFC (F5) (*r* = − 0.30, *p* = 0.04, one-tailed), but not for the right DLPFC stimulation condition (F6) (*r* = − 0.12, *p* = 0.25, one-tailed). After correction for two tests on the two hemispheres, a trend towards significance for the left stimulation condition remained (*r* = − 0.30, *p* = 0.07, one-tailed).

## Discussion

Examining N100 alterations longitudinally in adolescents with depression, we found significantly increased (i.e., more negative) N100 amplitudes in the DLPFC at baseline, coinciding with significantly more severe depressive symptoms. Thus, N100 amplitudes seem to be dependent on the depressive symptom severity in adolescents.

### N100 amplitudes and depression

Our results indicate altered cortical inhibition in the DLPFC depending on the depressive symptom severity in adolescents, as the N100 amplitude is considered to reflect GABA-B mediated cortical inhibition (Premoli et al. 2014; Rogasch et al. 2015). Higher N100 amplitude in a state with greater depression severity is congruent with cross-sectional greater N100 amplitudes in the DLPFC in (young) adults with MDD compared to healthy controls (Dhami et al. 2020; Voineskos et al. 2019). Longitudinal N100 amplitude decreases in association with an improvement in symptom severity have also been observed in adults, receiving six-week active rTMS stimulation (Voineskos et al. 2021). A trend towards a significant moderate correlation between reductions in N100 amplitude and depressive symptom severity in our data could indicate a clinically relevant association. The fact that the correlation was no longer significant after correction for multiple testing could be due to the large variability in difference values (T0–T1) between subjects, and the relationship might become more distinct in a larger sample.

Contrary, a lack of significant alterations in N100 amplitudes in young adults (16–24 years, *n* = 16) with MDD during a two-weeks theta burst intervention (Dhami et al. 2021) might argue against a dependency of N100 amplitudes on depression severity. Even though the N100 amplitude reductions were not significant, arguably due to the smaller sample size, descriptively larger N100 amplitudes at baseline were illustrated. That N100 changes reflect only order effects due to multiple measurements (T0 and T1) and not systematic changes as a function of depressive symptom severity seems unlikely, as TEPs are sensitive to changes in cortical properties as well as repeatable over time (Casarotto et al. 2010) and the N100 shows good reliability (Kerwin et al. 2018; Lioumis et al. 2009).

The found alterations in N100 amplitudes, indicating increased cortical inhibition, can be related to changes in synaptic transmission during a depressive episode. Increased prefrontal inhibition during a depressive episode has been explained in previous studies by an increased GABA turnover postsynaptically (Dhami et al. 2020; Voineskos et al. 2019) and/or an E/I imbalance, in which inhibition still outweighs excitation (Page and Coutellier 2019). Depression related deficits in GABA neurotransmitter levels (Duman et al. 2019; Hasler et al. 2007) might be over-compensated by increased activity of specific inhibitory GABAergic interneurons (Page and Coutellier 2019). The corresponding elevated interneuron activity might lead to increased inhibition and subsequently a hypoactivity of the prefrontal cortex, thus causing a higher TMS-EEG inhibitory N100 marker (Page and Coutellier 2019). This proposed aberrant E/I balance likely depends on the depression severity, as antidepressant effects of N-methyl-d-aspartate antagonist ketamine is caused by extensive glutamate release (Abdallah et al. 2018; Krystal et al. 2013; Lener et al. 2017) and other antidepressant therapies lead to increased cortical GABA levels during treatment (Bhagwagar et al. 2004; Dubin et al. 2016; Sanacora et al. 2003). N100 amplitude reductions during an antidepressant treatment therefore likely reflect ‘normalization’ of the E/I balance. The ‘normalization’ of the E/I imbalance and the antidepressant effect of our therapy could be due to an increase in GABA concentration, similar to other antidepressant therapies (Bhagwagar et al. 2004; Dubin et al. 2016; Sanacora et al. 2003). The normalization of the GABA concentration could in turn reverse the proposed over-compensatory activity of GABA-B interneurons. Thus, the reduced activity of GABA-B interneurons could lead to reduced hypoactivity in the DLPFC when the severity of depressive symptoms decreases. The reduced activity of GABA-B interneurons might be reflected in smaller N100 amplitudes after the intervention.

### Lateralized N100 amplitudes

To further investigate the reduction in N100 amplitudes between T0 and T1, the systematically lateralized activity to the stimulation side (left/right) was considered in more detail. TEP amplitudes have been shown to be systematically higher ipsilaterally than contralaterally, with the highest amplitudes at the stimulation site (Jarczok et al. 2021). A lateralized ipsilateral topography around the stimulation site is likely not only the result of peripheral stimulation (Conde et al. 2019) but rather transcranially evoked. The topographic maps of the original data (Fig. 1b) confirm a lateralized ipsilateral maximum at each stimulation site. To eliminate evoked activity that is not systematically lateralized at the stimulation site, we calculated LatTEP N100 amplitudes at the homologous electrodes of interest F5/F6. This process can reveal lateralized negativity at the site of stimulation that could be masked by symmetric processes in the original maps. The topographic maps of the LatTEP (Fig. 1c) show a negative maximum at the stimulation site (F5/F6) for T0 and T1 in the same time window as the original data (120–130 ms). Although the topographies indicate lower activity at T1 compared with T0, which is consistent with the amplitude reduction in the original N100 data, the LatTEP N100 reduction at F5/F6 did not surpass the threshold of statistical significance. The trend result could be due to the slightly smaller effect of depression severity on the lateralized part of N100 compared to the effect on the overall N100 (including lateralized and non-lateralized parts of the N100). Although the trend supports our findings on the original N100 alterations between time points, a larger sample is needed to further investigate the trend in lateralized potentials.

### N100 amplitudes and age

Exploring cortical inhibitory processes as a function of depressive symptom severity in various age groups seems crucial, as the prefrontal E/I balance in adolescents undergoes extensive maturational changes (Caballero and Tseng 2016; Kilb 2012; Page and Coutellier 2019). However, in this study no correlation between age and N100 amplitudes in the examined age-range was detected. Previous studies on cortical inhibition and age mainly focused on the motor cortex and reported age effects on N100 amplitudes in healthy participants of varying ages (Bender et al. 2005; Määttä et al. 2017). In 9–17-year-olds with depression a negative correlation between age and long-interval cortical inhibition (LICI), a GABA-B marker assessed by a double-pulse TMS-EEG paradigm, has also been reported (Croarkin et al. 2014). Age effects on GABA-B marker are probably driven by inclusion of pre-adolescent children as age effects were apparent when children were compared with adults and adolescents, but not when comparing adolescents to adults (Määttä et al. 2017). The lack of association between N100 and age in the investigated age group (12–18 years) is likely related to the small age variance in the analyzed sample.

### Hemispheric asymmetries

We found variations between hemispheres, as a trend towards significant correlation between difference values of N100 amplitudes and symptom severity was observed only during left DLPFC stimulation. For the left DLPFC, previous studies have reported correlations between baseline N100 amplitudes and changes in suicidal ideation (Sun et al. 2016) and correlations between N100 amplitudes and depressive symptoms (Voineskos et al. 2021, 2019).

Theories on anterior hemispheric asymmetries in depression have been discussed before, as EEG (Davidson et al. 2002; Miller et al. 2013) and fMRI (Grimm et al. 2008; Herrington et al. 2010) studies suggest reduced frontal activity in the left compared with the right hemisphere. Additionally, patients with MDD benefit from therapeutic high-frequency rTMS over the left DLPFC (increasing cortical activity) and low-frequency rTMS over the right DLPFC (suppressing cortical activity) (Chen et al. 2013; Gershon et al. 2003). Frontal left/right lateralization has been linked to positive/negative emotional valence processes (Davidson 1992; Heller et al. 1998) or approach/avoidance motivation (Davidson et al. 2002). The DLPFC seems to be involved in the cognitive regulation of emotional processing in the amygdala. Hypoactivity of the left DLPFC during a depressive episode might be associated with impaired downregulation of emotions with negative valence and impaired upregulation of positive emotions (Bruder et al. 2017). Normalization of over inhibition in the left DLPFC might represent a relevant antidepressant mechanism. However, we did not observe significant differences in N100 amplitudes between hemispheres, which would be expected if the left DLPFC is less active compared with the right DLPFC. Therefore, underlying mechanism and directionality need to be investigated in future studies to examine hemispheric differences in N100 amplitudes.

### Limitations

One limitation is that we did not use auditory masking. The TMS coil click results in an AEP reflected by a negative deflection at 100 ms and a positive peak 180–200 ms after the pulse (ter Braack et al. 2015). Thus, the measured N100 amplitude in our study reflects not only transcranially evoked cortical activity due to the TMS pulse but also, to some extent, peripherally evoked AEPs. However, it is important to consider the topography of the AEP-related N100 component, which has been described in central regions with bilateral distribution (Rogasch et al. 2014) or in the contralateral hemisphere during monaural acoustic stimulation (Hine and Debener 2007). A comparison between active and sham TMS stimulation conditions indicated that a N100 component with a topography over the vertex represents to a great extent a non-transcranial sensory evoked potential (Conde et al. 2019). As described previously, the lateralized ipsilateral topography of the N100 at the stimulation site (see Fig. 1) supports the notion that although the recorded N100 is affected by peripherally evoked AEPs to some extent, its ipsilateral amplitude reflects mainly transcranially evoked activity.

Despite an influence of the AEPs on the TEP N100, we believe that our results nevertheless allow valid conclusions to be drawn about intraindividual N100 changes, because stable AEP N100 components between measurements were described in intraindividual designs (Atcherson et al. 2006; Louzã et al. 1994). Moreover, the AEP N100 component seems to not be related systematically to depressive symptoms, as neither AEPs nor loudness dependency of AEPs differed between participants with and without MDD (Bruder et al. 1995; Feldmann et al. 2018; Park et al. 2010). The loudness dependent AEP at baseline measure predicts the response to antidepressive medication in patients with MDD, but the component does not change in longitudinal designs (Gallinat et al. 2000; Juckel et al. 2007; Lee et al. 2015). Although AEPs have a stable effect on N100 amplitudes, the measured N100 alterations are likely due to genuine depression related changes in the GABA-B system and not to AEP alterations.

Furthermore, TMS was perfomed without neuronavigation. Neuronavigation, although helpful during TMS coil positioning, significantly increases measurement time (Julkunen et al. 2009). Instead, coil positions were chosen based on the DLPFC localization method of Rusjan and colleagues (2010), which has been used previously in other studies (Cash et al. 2017; Fitzgerald et al. 2009; Noda et al. 2021; Rogasch et al. 2015; Sun et al. 2016).

## Conclusion

In conclusion, greater N100 amplitudes during a state of greater depression severity support the hypothesis of E/I imbalance in the DLPFC in adolescents, with over inhibition normalizing with decreasing depressive symptom severity. To our knowledge, the depressive symptom severity dependency of this inhibitory marker has been demonstrated for the first time in adolescents with MDD and may enable the future use of N100 amplitude as biomarker in the context of diagnostic and therapeutic assessments.

## Data Availability

The ethics committee did not grant permission to share study data with third parties or to upload data in anonymized form.

## Declarations

### Submission declaration

The manuscript has not been published previously and is not under consideration for publication elsewhere. All authors gave final approval of the version to be published and the manuscript, if accepted, will not be published elsewhere without the written consent of the copyright-holder.
